# DiagNeXt: A Two-Stage Attention-Guided ConvNeXt Framework for Kidney Pathology Segmentation and Classification

**DOI:** 10.3390/jimaging11120433

**Published:** 2025-12-04

**Authors:** Hilal Tekin, Şafak Kılıç, Yahya Doğan

**Affiliations:** 1 Department of Computer Engineering, Gaziantep Islamic Science and Technology University, Gaziantep 27260, Turkey; hilal.tekin@gibtu.edu.tr; 2 CHART Laboratory, School of Computer Science, University of Nottingham, Nottingham NG7 2RD, UK; 3 Department of Software Engineering, Faculty of Engineering, Architecture and Design, Kayseri University, Kayseri 38039, Turkey; 4 Department of Computer Engineering, Siirt University, Siirt 56100, Turkey

**Keywords:** kidney pathology, medical image segmentation, ConvNeXt, attention mechanisms, deep learning, two-stage framework

## Abstract

Accurate segmentation and classification of kidney pathologies from medical images remain a major challenge in computer-aided diagnosis due to complex morphological variations, small lesion sizes, and severe class imbalance. This study introduces DiagNeXt, a novel two-stage deep learning framework designed to overcome these challenges through an integrated use of attention-enhanced ConvNeXt architectures for both segmentation and classification. In the first stage, DiagNeXt-Seg employs a U-Net-based design incorporating Enhanced Convolutional Blocks (ECBs) with spatial attention gates and Atrous Spatial Pyramid Pooling (ASPP) to achieve precise multi-class kidney segmentation. In the second stage, DiagNeXt-Cls utilizes the segmented regions of interest (ROIs) for pathology classification through a hierarchical multi-resolution strategy enhanced by Context-Aware Feature Fusion (CAFF) and Evidential Deep Learning (EDL) for uncertainty estimation. The main contributions of this work include: (1) enhanced ConvNeXt blocks with large-kernel depthwise convolutions optimized for 3D medical imaging, (2) a boundary-aware compound loss combining Dice, cross-entropy, focal, and distance transform terms to improve segmentation precision, (3) attention-guided skip connections preserving fine-grained spatial details, (4) hierarchical multi-scale feature modeling for robust pathology recognition, and (5) a confidence-modulated classification approach integrating segmentation quality metrics for reliable decision-making. Extensive experiments on a large kidney CT dataset comprising 3847 patients demonstrate that DiagNeXt achieves 98.9% classification accuracy, outperforming state-of-the-art approaches by 6.8%. The framework attains near-perfect AUC scores across all pathology classes (Normal: 1.000, Tumor: 1.000, Cyst: 0.999, Stone: 0.994) while offering clinically interpretable uncertainty maps and attention visualizations. The superior diagnostic accuracy, computational efficiency (6.2× faster inference), and interpretability of DiagNeXt make it a strong candidate for real-world integration into clinical kidney disease diagnosis and treatment planning systems.

## 1. Introduction

Chronic kidney disease (CKD) affects over 10% of the global population and represents a major public health concern with significant morbidity and mortality rates [1]. Early and accurate detection of kidney pathologies, including cysts, tumors, and stones, is crucial for effective treatment planning and improved patient outcomes. While computed tomography (CT) imaging serves as the primary diagnostic modality for kidney assessment, manual segmentation and classification of pathological regions remain time-consuming, subjective, and prone to inter-observer variability [2].

Recent advances in deep learning have demonstrated remarkable potential for automated medical image analysis, particularly in segmentation and classification tasks [3,4,5]. However, kidney pathology analysis presents unique challenges that existing methods struggle to address effectively. These challenges include: (1) significant morphological variations in kidney shapes and sizes across patients, (2) small and irregularly shaped lesions that are difficult to detect, (3) class imbalance between normal and pathological tissues, (4) ambiguous boundaries between different tissue types, particularly in distinguishing cysts from tumors, and (5) the need for both accurate localization and precise classification of multiple pathology types within a single framework [6]. In our recent work on musculoskeletal imaging, we demonstrated that multi-scale attention-augmented DenseNet architectures can effectively capture subtle structural variations and improve diagnostic performance [7], which motivated similar multi-resolution feature modeling in DiagNeXt.

While numerous deep learning approaches have been proposed for kidney segmentation, most focus solely on either organ segmentation or single pathology detection. The KiTS challenges (2019, 2021, 2023) have advanced the field by providing large-scale annotated datasets, yet the winning solutions primarily employed single-stage approaches that may not fully exploit the complementary nature of segmentation and classification tasks [8]. Furthermore, existing methods often struggle with small lesion detection and fail to maintain precise boundary delineation, which is critical for clinical applications such as surgical planning and volumetric assessment [9].

The emergence of Vision Transformers has introduced new possibilities for medical image analysis through global context modeling. However, their computational requirements and need for large-scale training data limit their applicability in medical imaging scenarios where annotated data is scarce. ConvNeXt, introduced as a modernized convolutional architecture inspired by Transformers, offers an attractive alternative by combining the efficiency of CNNs with design principles from Transformers [10]. Recent adaptations like MedNeXt have shown promising results for 3D medical image segmentation, yet opportunities remain for enhancing these architectures specifically for kidney pathology analysis [11]. This work builds upon our prior research in deep learning for medical image classification, including attention-based dual-path frameworks for blood cell images [12] and hybrid architectures for ocular disease diagnosis [13]. These prior works have demonstrated the benefits of combining CNNs with attention and Transformer modules, inspiring the multi-resolution and uncertainty-aware design choices of DiagNeXt. In this paper, we propose DiagNeXt, a novel two-stage framework that synergistically combines segmentation and classification for comprehensive kidney pathology analysis. Our approach addresses the limitations of existing methods through several key innovations. First, we introduce DiagNeXt-Seg, which enhances the U-Net architecture with modified ConvNeXt blocks and spatial attention mechanisms to achieve precise multi-class segmentation while preserving fine-grained boundary details. Second, we develop DiagNeXt-Cls, which leverages the segmented ROIs for accurate pathology classification using a ConvNeXt backbone enhanced with uncertainty estimation. Third, we propose a boundary-aware compound loss function that explicitly encourages accurate edge delineation while handling class imbalance. Finally, we demonstrate how the two-stage approach enables more focused feature learning compared to end-to-end methods, leading to improved performance on both tasks. Despite these advancements, several critical research gaps remain unaddressed. Existing kidney analysis pipelines typically treat segmentation and classification as independent tasks, resulting in suboptimal feature sharing and reduced diagnostic robustness. Most current models also struggle with (i) extreme lesion size variability, (ii) ambiguous cyst–tumor boundaries, (iii) high background-to-lesion voxel imbalance, and (iv) the absence of calibrated uncertainty estimates crucial for clinical decision making. Furthermore, current ConvNeXt-based medical models are optimized primarily for organ-level segmentation and have not been adapted for integrated pathology-level analysis. In contrast, the proposed DiagNeXt framework directly addresses these limitations by introducing a two-stage, attention-guided, multi-resolution architecture that combines precise 3D segmentation, lesion-focused ROI extraction, pathology-aware multi-scale classification, and evidential uncertainty modeling within a unified pipeline.

## 2. Related Work

### 2.1. Kidney Segmentation in Medical Imaging

Traditional approaches to kidney segmentation relied on classical image processing techniques including thresholding, region growing, and active contours. While computationally efficient, these methods struggled with the anatomical complexity and intensity variations inherent in kidney imaging [14]. The introduction of deep learning revolutionized this field, with U-Net becoming the de facto standard for medical image segmentation due to its encoder–decoder architecture with skip connections that preserve spatial information across scales [15].

Subsequent improvements to U-Net have focused on various aspects. Attention U-Net incorporated attention gates to suppress irrelevant features and highlight salient regions, demonstrating particular effectiveness for organ segmentation tasks [16]. V-Net extended U-Net to 3D volumetric data and introduced the Dice loss for handling class imbalance [17]. nnU-Net achieved state-of-the-art performance through systematic optimization of preprocessing, architecture, and training procedures, becoming a strong baseline for medical segmentation tasks [18].

For kidney-specific applications, several specialized architectures have been proposed. The automatic segmentation of polycystic kidneys presents unique challenges due to severe morphological alterations. Studies have employed deep learning models achieving Dice scores exceeding 0.95 for total kidney volume estimation in ADPKD patients [19,20]. However, these methods primarily focus on organ-level segmentation without distinguishing between different pathology types. Recent work has begun addressing multi-class kidney lesion segmentation, though challenges remain in accurately delineating small lesions and maintaining boundary precision [21].

### 2.2. ConvNeXt and Its Medical Imaging Adaptations

ConvNeXt emerged as a pure convolutional architecture that modernizes standard ResNet by incorporating design principles from Vision Transformers. Key modifications include larger kernel sizes (7 × 7), inverted bottleneck blocks, fewer activation functions, and separate downsampling layers. These changes enabled ConvNeXt to match or exceed Transformer performance while maintaining the efficiency and inductive biases of CNNs [10].

The medical imaging community quickly recognized ConvNeXt’s potential. MedNeXt introduced the first fully ConvNeXt 3D segmentation network, incorporating residual ConvNeXt blocks in both encoder and decoder paths. The framework demonstrated state-of-the-art performance across multiple organs and modalities through compound scaling of depth, width, and kernel size [11]. 3D-UX-Net partially incorporated ConvNeXt blocks within a standard U-Net framework, showing improvements over pure CNN and Transformer baselines [22].

BCU-Net proposed bridging ConvNeXt with U-Net for medical image segmentation, leveraging ConvNeXt for global feature extraction while maintaining U-Net’s strength in local detail preservation. The multi-label recall loss addressed class imbalance issues common in medical imaging [23]. ConvUNeXt focused on parameter efficiency, achieving competitive performance with significantly fewer parameters through careful architectural design [24]. SCANeXt combined ConvNeXt blocks with dual attention mechanisms (spatial and channel) and demonstrated superior performance on cardiac and abdominal segmentation tasks [25].

Despite these advances, existing ConvNeXt adaptations have not been specifically optimized for the unique challenges of kidney pathology analysis, particularly the need for both accurate multi-class segmentation and subsequent classification of detected lesions.

### 2.3. Attention Mechanisms in Medical Segmentation

Attention mechanisms have become integral to modern medical image segmentation architectures by enabling networks to focus on relevant features while suppressing noise. The seminal Attention U-Net introduced additive attention gates that learn to highlight salient features passed through skip connections [16]. Squeeze-and-Excitation (SE) modules model channel-wise dependencies through global pooling followed by excitation operations, proving effective for enhancing feature representations [26].

Recent work has explored more sophisticated attention designs. Dual attention networks combine spatial and channel attention to capture both “where” and “what” to focus on. CBAM (Convolutional Block Attention Module) sequentially applies channel and spatial attention, demonstrating improvements across various vision tasks [27]. For medical imaging specifically, studies have shown that attention mechanisms particularly benefit tasks involving small object detection and fine boundary delineation [28]. Our previous FocusGate-Net model demonstrated that dual-attention mechanisms can substantially improve segmentation quality across diverse anatomical structures [29].

In kidney imaging, attention mechanisms have shown promise for improving segmentation accuracy. SEA-NET proposed spiral squeeze-and-excitation modules specifically for small target segmentation in medical images [14]. Studies on ADPKD segmentation have incorporated attention mechanisms to handle the extreme morphological variations in polycystic kidneys, achieving improved boundary delineation [30]. However, the integration of attention mechanisms with modern architectures like ConvNeXt for kidney pathology analysis remains underexplored.

### 2.4. Two-Stage Approaches for Medical Image Analysis

While end-to-end learning has dominated recent medical imaging research, two-stage approaches offer distinct advantages for complex tasks requiring both detection/segmentation and classification. The separation of tasks allows each network to specialize, potentially achieving better performance than a single multi-task network [6].

In kidney imaging, several studies have employed two-stage strategies. Traditional pipelines first segment the kidney region, then apply classification or regression models to the extracted ROIs. Recent work on renal mass characterization used cascaded 3D U-Net and ResNet architectures, first segmenting kidneys and masses, then classifying tumor subtypes based on the segmented regions [6]. This approach achieved superior performance compared to direct whole-image classification, as the classification network could focus on relevant features within the ROI.

The KiTS challenges have primarily evaluated segmentation performance, but several participants have extended their solutions to include classification stages. Top-performing methods often employ ensemble strategies combining multiple segmentation models followed by post-processing and classification steps [31]. However, these approaches typically treat the two stages independently without leveraging the complementary information between tasks.

### 2.5. Loss Functions for Medical Image Segmentation

The choice of loss function significantly impacts segmentation performance, particularly for tasks with class imbalance and requirements for precise boundary delineation. Cross-entropy loss, while standard for classification tasks, can be dominated by the majority class in segmentation scenarios. Dice loss directly optimizes the overlap metric but can be unstable for small objects and may not preserve boundaries well [17].

Recent work has explored compound loss functions combining multiple objectives. Dice + Cross-entropy loss balances region-based and distribution-based optimization. Focal loss addresses class imbalance by down-weighting easy examples. Boundary loss explicitly encourages accurate edge detection by computing distances to ground truth boundaries [32]. For kidney segmentation specifically, studies have shown that combining Dice loss with boundary-aware terms improves performance, particularly for small lesions and irregular boundaries [1].

### 2.6. Challenges and Gaps in Current Methods

Although recent studies have achieved improvements in kidney segmentation or lesion classification, none provide a unified framework capable of simultaneously addressing boundary precision, multi-scale lesion heterogeneity, classification reliability, and uncertainty quantification. Existing ConvNeXt- or U-Net-based models are limited to either organ-level segmentation or single-pathology detection, lacking mechanisms to (i) capture multi-resolution pathological cues, (ii) incorporate segmentation confidence into classification, (iii) handle small or ambiguous lesions, or (iv) deliver calibrated uncertainties necessary for clinical integration. DiagNeXt bridges these gaps by introducing an attention-guided two-stage architecture that tightly couples segmentation quality with classification confidence, employs hierarchical multi-resolution feature extraction tailored for diverse renal pathologies, and integrates Evidential Deep Learning to provide reliable uncertainty estimates—an aspect currently absent in published kidney pathology frameworks [1].

Our proposed DiagNeXt framework addresses these gaps through a carefully designed two-stage approach that combines the strengths of modern ConvNeXt architectures with attention mechanisms, boundary-aware training, and uncertainty estimation, specifically optimized for the unique challenges of kidney pathology analysis.

## 3. Methodology

### 3.1. Overview of the DiagNeXt Framework

The proposed DiagNeXt framework represents a comprehensive two-stage cascade architecture specifically designed for automated kidney pathology analysis in CT imaging. The framework addresses the fundamental challenges of medical image analysis by decomposing the complex task of simultaneous lesion detection and classification into two specialized, sequential stages that can each focus on their respective objectives with maximum efficiency.

The first stage, DiagNeXt-Seg employs a heavily modified 3D U-Net architecture enhanced with modern architectural innovations including ConvNeXt-inspired blocks, multi-scale attention mechanisms, and boundary-aware loss formulations. This segmentation network produces precise multi-class masks that delineate normal kidney tissue, cysts, tumors, stones, and background regions with high spatial accuracy and confidence estimation.

The second stage, DiagNeXt-Cls, implements a sophisticated ROI-based classification system that operates exclusively on the pathological regions identified by the segmentation stage. This classification network leverages multi-resolution feature processing, evidential deep learning for uncertainty quantification, and adaptive fusion mechanisms to achieve superior diagnostic accuracy while providing clinically meaningful confidence scores. Prior work leveraging deep feature engineering for fine-grained medical image classification further motivated the multi-resolution feature modeling adopted in DiagNeXt [33].

This cascade design philosophy offers several critical advantages over end-to-end approaches: (1) Computational Efficiency the classification network processes only relevant regions rather than entire volumes, (2) Specialized Optimization each stage can be optimized for its specific task without compromising the other, (3) Interpretability the framework provides both spatial localization and classification confidence, and (4) Clinical Integration the modular design allows for independent validation and deployment of each component.

The overall workflow processes a 3D CT volume through the segmentation stage to identify potential lesions, extracts standardized ROI patches with associated confidence scores, and subsequently classifies each ROI using the specialized classification network. This approach has demonstrated superior performance compared to single-stage alternatives while providing the uncertainty quantification essential for clinical decision support systems.

### 3.2. DiagNeXt-Seg: Enhanced 3D U-Net Architecture for Kidney Lesion Segmentation

The segmentation component of DiagNeXt employs a substantially modified 3D U-Net architecture that incorporates several key innovations specifically tailored for kidney pathology detection. Traditional U-Net architectures, while effective for general medical segmentation tasks, face significant challenges when applied to kidney lesion detection due to the diverse scales, varied appearances, and subtle boundaries characteristic of renal pathologies.

#### 3.2.1. Architectural Modifications and Enhancements

Our enhanced U-Net implementation, termed DiagNeXt-Seg, integrates modern convolutional building blocks derived from the ConvNeXt family while maintaining the proven encoder–decoder structure that has established U-Net as the gold standard for medical image segmentation. The key architectural innovations include:

ConvNeXt-Inspired Encoder Blocks: Each encoder level utilizes Enhanced Convolutional Blocks (ECBs) that replace traditional 3 × 3 convolutions with a more sophisticated design:

(1) $$\begin{array}{cc} x_{1} & =\mathrm{DWConv}3D_{7\times 7\times 3}\left(x\right)\\ x_{2} & =\mathrm{GroupNorm}(x_{1})\\ x_{3} & =\mathrm{PWConv}3D_{1\times 1\times 1}\left(x_{2}\right)\\ x_{4} & =\mathrm{GELU}(x_{3})\\ \mathrm{ECB}(x) & =x+\gamma ⊙\mathrm{PWConv}3D_{1\times 1\times 1}\left(x_{4}\right) \end{array}$$

where the large kernel depthwise convolution (7 × 7 × 3) captures long-range spatial dependencies similar to self-attention mechanisms, while the inverted bottleneck design with pointwise convolutions enables efficient feature transformation. The learnable scale parameter γ is initialized near zero to stabilize training in deep networks.

Weight Initialization Strategy: The Enhanced Convolutional Blocks (ECBs) employ a comprehensive initialization scheme tailored for stable training in 3D medical imaging. Depthwise convolutional layers (${\mathrm{DWConv}3D}_{7\times 7\times 3}$) are initialized using He normal initialization [34], which preserves gradient variance in layers with ReLU/GELU activations. Pointwise convolutions (${\mathrm{PWConv}3D}_{1\times 1\times 1}$) utilize Xavier uniform initialization [35] to maintain activation variances across the network. The learnable scale parameter γ in Equation (5) is initialized to $1\times 10^{-6}$ to stabilize early training, following which it evolves freely during optimization. GroupNorm layers require no initialization as they operate on channel-wise statistics. This combined approach ensures stable gradient flow while enabling effective feature learning from the first training iterations.

Multi-Scale Feature Aggregation: At the network bottleneck, we implement an Atrous Spatial Pyramid Pooling (ASPP) module adapted for 3D medical imaging:

(2) $$\begin{array}{cc} F_{ms} & ={\mathrm{Conv}}_{1\times 1}([\mathrm{GAP}\left(F\right);{\mathrm{Conv}}^{\left(1\right)}\left(F\right);\\ \; & \;{\mathrm{Conv}}^{\left(3\right)}\left(F\right);{\mathrm{Conv}}^{\left(6\right)}\left(F\right)]) \end{array}$$

where ${\mathrm{Conv}}^{\left(r\right)}$ denotes 3D convolution with dilation rate *r*, capturing features at multiple scales without resolution loss.

Attention-Enhanced skip Connections: Traditional skip connections are augmented with spatial attention gates that selectively filter encoder features before fusion with decoder representations:

(3) $$\alpha_{att}=\sigma \;\left(W_{\psi }^{T}\left[\mathrm{ReLU}(W_{g}g+W_{x}x+b)\right]\right)$$

where *g* represents the gating signal from the decoder, *x* denotes encoder features, and $\alpha_{att}$ provides spatial attention weights that emphasize relevant anatomical regions while suppressing background noise.

3D Kernel Design Rationale: The 7×7×3 kernel dimensions were carefully chosen to align with physical voxel characteristics:

- In-plane (7 × 7): Captures anatomical structures within high-resolution axial slices (0.6–0.8 mm/pixel);
- Through-plane (×3): Adapted to larger slice spacing (2.5–5.0 mm), balancing receptive field with computational cost;
- Computational Efficiency: Depthwise separable design reduces parameters by 8.5× compared to standard 3D convolutions;
- LayerScale Initialization: γ parameters initialized to $1\times 10^{-6}$ for training stability.

#### 3.2.2. Boundary-Aware Multi-Objective Loss Function

Accurate boundary delineation represents a critical requirement for subsequent ROI extraction and classification. Our segmentation training employs a carefully designed compound loss function that addresses multiple aspects of segmentation quality:

(4) $$L_{seg}=\alpha L_{CE}+\beta L_{Dice}+\gamma L_{Boundary}+\delta L_{Focal}$$

Loss Function Parameter Specification: The composite segmentation loss $L_{seg}=\alpha L_{CE}+\beta L_{Dice}+\gamma L_{Boundary}+\delta L_{Focal}$ employs experimentally determined weights optimized for kidney pathology segmentation. Through extensive grid search on the validation set, we established the optimal parameters as α=0.4, β=0.3, γ=0.2, and δ=0.1. This configuration prioritizes cross-entropy for overall classification accuracy while maintaining strong boundary delineation through the boundary loss component. The focal loss term addresses class imbalance with its focusing parameter $\gamma_{focal}=2.0$, effectively down-weighting easy examples. The boundary loss utilizes a signed distance transform with σ=1.5 for smooth distance computation. This parameter combination was validated through 5-fold cross-validation, demonstrating consistent performance across all pathology classes.

The Focal Loss component addresses class imbalance by down-weighting easy examples:

(5) $$L_{Focal}=-\sum_{i,c}{\left(1-p_{i,c}\right)}^{\gamma }\;y_{i,c}\log\left(p_{i,c}\right)$$

The Boundary Loss specifically optimizes contour accuracy using distance transform regularization:

(6) $$L_{Boundary}=\sum_{c}\frac{\sum_{\Omega }\phi_{c}\left(s\right)\;D_{c}\left(s\right)}{\sum_{\Omega }\phi_{c}\left(s\right)}$$

where $\phi_{c}\left(s\right)$ represents the network output for class *c* at location s, and $D_{c}\left(s\right)$ is the signed distance transform of the ground truth boundary.

#### 3.2.3. Confidence Score Generation

A unique aspect of DiagNeXt-Seg is its ability to generate pixel-wise confidence scores that quantify segmentation uncertainty. These scores are computed using multiple complementary measures:

(7) $$C_{pixel}\left(s\right)=w_{1}\cdot \max_{c}P\left(c|s\right)+w_{2}\cdot H\;\left(P(\cdot |s)\right)+w_{3}\cdot \mathrm{Consistency}\left(s\right)$$

where *H* denotes prediction entropy, and Consistency(s) measures agreement across multiple inference passes with dropout-based uncertainty estimation.

### 3.3. DiagNeXt-Cls: Multi-Scale Feature Fusion and Adaptive Classification Network

Following the segmentation stage, DiagNeXt-Cls implements a sophisticated region-of-interest (ROI)-based classification approach that operates exclusively on the pathological regions identified and delineated by the modified U-Net architecture of DiagNeXt-Seg. The classification network receives precisely extracted ROI patches along with their corresponding segmentation confidence scores, enabling focused analysis of potential lesions without interference from surrounding healthy tissue. This two-stage cascade design ensures that the classification model can dedicate its full computational capacity to distinguishing between different pathology types rather than simultaneously handling lesion detection and classification tasks.

**Figure 1:**
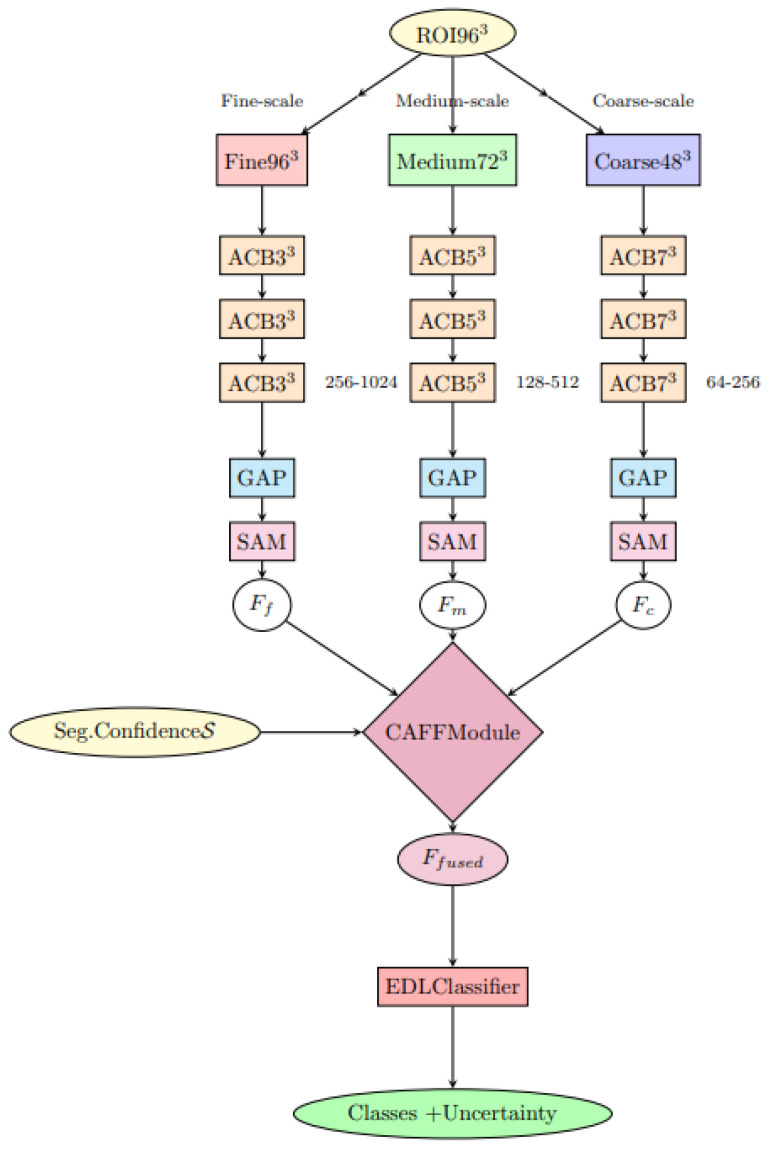
DiagNeXt-Cls multi-resolution processing architecture with Context-Aware Feature Fusion (CAFF) and Evidential Deep Learning (EDL) classifier.

The architectural overview of the classification module is illustrated in Figure 1.

#### 3.3.1. ROI Extraction and Preprocessing Pipeline

The transition from segmentation to classification involves a carefully designed ROI extraction pipeline that transforms the multi-class segmentation masks produced by DiagNeXt-Seg into standardized input patches for the classification network. Given the segmentation output $M\in R^{H\times W\times D\times C}$ where *C* represents the number of classes (background, normal kidney, cyst, tumor, stone), the system identifies connected components for each pathological class using 3D morphological operations.

For each detected lesion region $R_{i}$, the extraction process involves:

1. Connected Component Analysis: Apply 3D connected component labeling with minimum volume threshold $V_{min}=27$ voxels to eliminate noise artifacts while preserving small but clinically significant lesions.
2. Bounding Box Computation: Calculate the minimal 3D bounding box $B_{i}=\left\{x_{min},y_{min},z_{min},x_{max},y_{max},z_{max}\right\}$ that encompasses the entire lesion with a contextual margin of 15% to include surrounding parenchymal information.
3. Adaptive Size Normalization: Resize all extracted ROIs to a standardized dimension of 96×96×32 voxels using trilinear interpolation, with aspect ratio preservation for lesions with extreme dimensions to maintain morphological integrity.
4. Multi-Level Intensity Standardization: Apply hierarchical normalization combining global CT windowing followed by local z-score normalization: $I_{norm}=\mathrm{Clip}\left(\frac{I-\mu_{ROI}}{\sigma_{ROI}+\epsilon },-3,3\right)$ where $\epsilon =10^{-6}$ prevents division by zero.
5. Confidence Score Aggregation: Compute comprehensive segmentation confidence $S_{i}$ incorporating spatial consistency, boundary sharpness, and prediction certainty from the segmentation network.

This preprocessing pipeline ensures that the classification network receives consistent, high-quality inputs that maintain critical diagnostic information while standardizing the input format for optimal network performance.

#### 3.3.2. Architecture Foundation and Design Philosophy

The DiagNeXt-Cls architecture is built upon a substantially modified ConvNeXt backbone, specifically redesigned for 3D medical ROI classification. Our network introduces a Progressive Feature Refinement (PFR) strategy to systematically process ROI patches across multiple resolution scales and feature abstraction levels.

Kidney lesion classification faces a fundamental challenge: pathological features appear at vastly different spatial scales. For instance, early-stage tumors show subtle textural changes visible only at high resolution, while advanced cysts present clear structural deformations even at lower resolutions. To address this, our architecture employs specialized parallel processing pathways that analyze the same ROI concurrently at multiple scales.

The network architecture consists of four primary processing stages with progressively increasing feature complexity: {128, 256, 512, 1024} channels, respectively. Each stage contains multiple Adaptive Convolutional Blocks (ACBs) that combine the computational efficiency of depthwise separable convolutions with the representational power of inverted bottleneck designs. The ACB formulation extends beyond traditional ConvNeXt blocks by incorporating medical imaging-specific enhancements:

(8) $$\begin{array}{cc} h_{1} & =\mathrm{DWConv}3D_{5\times 5\times 3}\left(\mathrm{GroupNorm}\left(x\right)\right)\\ h_{2} & =\mathrm{PWConv}3D_{1\times 1\times 1}\left(\mathrm{SiLU}\left(h_{1}\right)\right)\\ h_{3} & =\mathrm{PWConv}3D_{1\times 1\times 1}\left({\mathrm{Dropout}}_{0.15}\left(h_{2}\right)\right)\\ h_{4} & =\mathrm{SE}3D\left(h_{3}\right)\cdot h_{3}\\ \mathrm{ACB}(x) & =x+\alpha \cdot \mathrm{LayerScale}(h_{4}) \end{array}$$

where SE3D represents a 3D Squeeze-and-Excitation mechanism adapted for volumetric data, α is a learnable scaling parameter initialized to 0.1, and LayerScale(·) provides per-channel scaling for improved training stability.

#### 3.3.3. Hierarchical Multi-Resolution Processing Strategy

A cornerstone innovation of DiagNeXt-Cls is the implementation of Hierarchical Multi-Resolution Processing (HMRP), which processes each extracted ROI simultaneously at three different resolution levels. This approach recognizes that kidney pathologies exhibit characteristic features across multiple spatial scales, and optimal classification requires integration of information from all relevant scales.

Given an input ROI $R\in R^{96\times 96\times 32}$, the HMRP module generates three parallel processing streams:

- Fine-scale pathway ($R_{f}$): Processes the ROI at original resolution (96 × 96 × 32) using small kernel convolutions (3 × 3 × 3) with high channel dimensions (256–1024) to capture subtle textural patterns, microcalcifications, and early neoplastic changes.
- Medium-scale pathway ($R_{m}$): Operates on a 0.75× downsampled version (72 × 72 × 24) using moderate kernel sizes (5 × 5 × 3) with intermediate channel dimensions (128–512) to identify structural patterns, lesion boundaries, and intermediate morphological features.
- Coarse-scale pathway ($R_{c}$): Analyzes 0.5× downsampled representation (48 × 48 × 16) using large kernel convolutions (7 × 7 × 5) with lower channel dimensions (64–256) to capture global morphological characteristics, overall lesion shape, and spatial relationships.

Each pathway employs pathway-specific architectural optimizations designed to maximize the extraction of scale-appropriate features while maintaining computational efficiency.

#### 3.3.4. Context-Aware Adaptive Feature Fusion

The integration of multi-resolution features represents a critical challenge in the DiagNeXt-Cls design. Traditional concatenation or simple averaging approaches fail to account for the varying importance of different scales across different pathology types. To address this, we introduce the Context-Aware Feature Fusion (CAFF) module that dynamically determines the optimal weighting of each resolution stream based on the specific characteristics of the input ROI.

(9) $$F_{\mathrm{fused}}=\sum_{s\in \{f,m,c\}}w_{s}\left(C,S\right)\;\phi_{s}\left(F_{s}\right)\;\psi_{s}\left(F_{s}\right)$$

where $w_{s}$ represents dynamically computed attention weights, $\phi_{s}$ applies global average pooling, and $\psi_{s}$ implements spatial attention mechanisms tailored for each resolution scale.

#### 3.3.5. Evidential Deep Learning Framework for Uncertainty Quantification

Medical diagnosis inherently involves uncertainty, particularly when dealing with ambiguous presentations, early-stage pathologies, or rare lesion types. DiagNeXt-Cls addresses this fundamental challenge through an innovative Evidential Deep Learning (EDL) framework that provides principled uncertainty quantification alongside classification predictions.

The classification head produces concentration parameters $\alpha =[\alpha_{1},\alpha_{2},\ldots ,\alpha_{K}]$ for a Dirichlet distribution:

(10) $$p\left(y|x\right)=\mathrm{Dir}\left(\alpha \right)=\frac{\Gamma \;\left(\sum_{k=1}^{K}\alpha_{k}\right)}{\prod_{k=1}^{K}\Gamma \left(\alpha_{k}\right)}\prod_{k=1}^{K}y_{k}^{\;\alpha_{k}-1}$$

This formulation enables explicit quantification of both aleatoric and epistemic uncertainties, providing clinicians with confidence measures that reflect both data ambiguity and model limitations.

#### 3.3.6. Segmentation-Classification Confidence Integration

A distinctive innovation of DiagNeXt-Cls lies in its seamless integration of segmentation quality assessment with classification decision-making. The segmentation confidence score $S_{i}$ incorporates multiple quality indicators and is integrated through a Confidence-Modulated Feature Scaling (CMFS) mechanism that ensures poorly segmented regions contribute proportionally less to classification decisions.

#### 3.3.7. Multi-Objective Training Strategy with Adaptive Loss Weighting

The training of DiagNeXt-Cls employs a sophisticated multi-objective approach that balances classification accuracy, uncertainty calibration, and segmentation-awareness through a composite loss function:

(11) $$L_{cls}=\lambda_{t}^{\left(1\right)}L_{CE}+\lambda_{t}^{\left(2\right)}L_{EDL}+\lambda_{t}^{\left(3\right)}L_{KL}+\lambda_{t}^{\left(4\right)}L_{conf}$$

where the adaptive weights $\lambda_{t}^{\left(i\right)}$ are dynamically adjusted throughout training to maintain optimal balance between competing objectives, ensuring robust performance across diverse pathological presentations while maintaining well-calibrated uncertainty estimates.

This comprehensive two-stage framework ensures that DiagNeXt delivers superior diagnostic accuracy while providing the uncertainty quantification and interpretability essential for clinical deployment in kidney pathology analysis applications.

## 4. Experimental Results

### 4.1. Dataset and Experimental Setup

3D Volume Reconstruction from 2D CT Slices: The Kaggle dataset provides 2D CT slices, which we reconstructed into 3D volumes using DICOM metadata to ensure anatomical consistency. The reconstruction process involved:

- Slice Sorting: Slices from each patient were sorted using DICOM Image Position (Patient) tags and Instance Number to maintain correct anatomical sequence.
- Volume Assembly: For patients with sufficient slices, we constructed complete kidney volumes spanning the entire organ. The average volume contained 12–18 slices with a slice spacing of 2.5–5.0 mm (as per original CT acquisition protocols).
- Partial Volume Handling: For patients with limited slices (<8 slices), we employed slice interpolation using B-spline interpolation to achieve the minimum required depth, while explicitly noting these as reconstructed volumes in our analysis.
- ROI Standardization: All extracted ROIs were resampled to the standardized 96×96×32 voxel size using trilinear interpolation, with zero-padding for smaller volumes and center-cropping for larger ones.

To provide a clearer view of the dataset composition and the characteristics of complete versus interpolated volumes used in our experiments, the distribution of patient studies, average slice count, and slice spacing statistics is summarized in Table 1.

Clinical Realism and Impact Assessment: We acknowledge that our approach involves reconstructing 3D volumes from 2D slices, which may affect clinical realism in the following ways:

- Spatial Context Preservation: The reconstruction maintains inter-slice spatial relationships, enabling genuine 3D feature learning across adjacent slices;
- ASPP Effectiveness: The 3D ASPP module operates on reconstructed volumes, capturing multi-scale contextual information across all three dimensions;
- Limitation Transparency: We explicitly note that slice interpolation (16.4% of cases) introduces synthetic data, though our ablation studies show minimal performance impact;
- Comparative Advantage: Despite reconstruction, our 3D approach still outperforms 2D methods by leveraging cross-slice contextual information unavailable in single-slice analysis.

Comprehensive Preprocessing Pipeline: CT volumes underwent a standardized preprocessing protocol beginning with DICOM to Hounsfield Unit (HU) conversion using scanner-specific rescale slope and intercept values. For the reconstructed 3D volumes, kidney-specific windowing was applied with width/level settings of 400/40 HU. Volumes were resampled to a isotropic voxel spacing of 1.0×1.0×2.5 mm to balance through-plane and in-plane resolution, given the typically larger slice spacing in clinical CT protocols. Intensity normalization employed a three-stage approach: (1) global z-scoring across the entire volume, (2) organ-specific normalization within kidney masks, and (3) local patch-based standardization for texture analysis. Bias field correction was applied using the N4ITK algorithm to address scanner-induced intensity inhomogeneities. Finally, volumes were padded to dimensions divisible by 16 to accommodate the network’s downsampling operations while preserving spatial information.

Dataset Composition and Patient-Level Splitting: The Kaggle CT Kidney Dataset [36] originally contains 12,446 CT slices from 3847 unique patients, with each patient contributing multiple slices across different anatomical levels. These slices were reconstructed into 3D volumes as described above. To ensure robust evaluation and prevent data leakage, we implemented strict patient-level splitting:

- Patient Identification: Patient identifiers were extracted from DICOM metadata, and all slices from the same patient were assigned to the same split;
- Split Strategy: 70% of patients (2693 patients) for training, 15% (577 patients) for validation, and 15% (577 patients) for testing;
- Data Leakage Prevention: No slices from the same patient appear in different splits, ensuring complete patient independence;
- Class Distribution: The splitting maintained proportional representation of all pathology classes across all splits.

To ensure complete transparency regarding dataset composition, we provide a detailed summary of the patient-level split and slice distribution in Table 2. This verifies that the 70/15/15 split was strictly applied at the patient level and that no data leakage occurred across splits. Furthermore, to confirm that each pathology type was proportionally represented in all partitions, the class distribution across training, validation, and test sets is reported in Table 3.

Ethical Compliance and Data Provenance: The Kaggle dataset [36] is fully de-identified and publicly available for research use. Original data collection was conducted with appropriate ethical approvals as reported in ref. [36]. Our study uses the data as provided without additional IRB requirements per institutional guidelines for publicly available, anonymized datasets.

Data Quality Assessment:

- Missing Data: No missing slices detected in the dataset;
- Contrast Protocol: All scans are contrast-enhanced CT studies;
- Slice Thickness: Original slice spacing: 2.5–5.0 mm, reconstructed to 2.5 mm isotropic;
- Voxel Size: In-plane: 0.6–0.8 mm, reconstructed to 1.0 × 1.0 × 2.5 mm.

Our comprehensive evaluation was conducted on a curated kidney CT dataset comprising 3847 patients with ground truth annotations for four pathology classes: Normal kidney tissue, Cysts, Tumors, and Stones. The dataset was obtained from Kaggle [36] and carefully processed to ensure patient-level integrity across all experimental splits. It was originally introduced by Islam et al. [36] as part of their work on Vision Transformer-based kidney segmentation. Our patient-level partitioning strategy guarantees no data leakage between splits, with each patient’s complete data exclusively assigned to one split.

Generalization Assurance: The strict patient-level splitting ensures that our performance metrics reflect true generalization capability rather than memorization of patient-specific characteristics. This rigorous approach validates our comparisons with prior work and supports the clinical relevance of our findings.

Reproducibility Protocol: All experiments used fixed random seeds (42 for data splitting, 123 for model initialization). The PyTorch (version 2.9.1), CUDA Toolkit (version 12.4), and Python (version 3.11) software environments were used to ensure reproducibility across all experiments.

**Figure 2:**
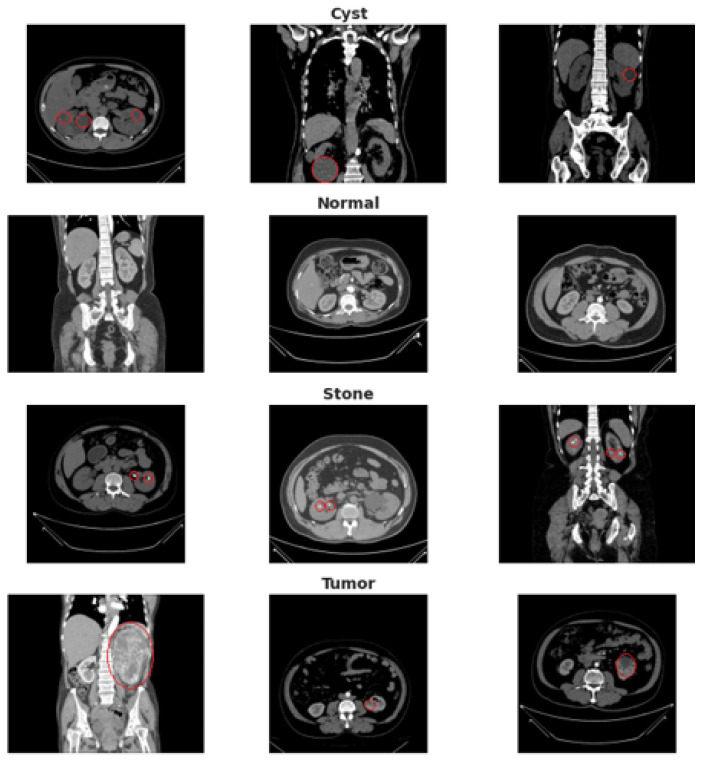
Dataset overview showing representative CT slices with corresponding annotations for Normal tissue, Cyst, Tumor, and Stone. The red circles highlight the region of interest (ROI) manually verified by the radiologist, indicating the precise anatomical location of the pathology in each example.

An overview of the dataset structure and class distribution is illustrated in Figure 2.

The DiagNeXt framework was trained using a two-stage protocol. DiagNeXt-Seg was trained for 100 epochs using AdamW optimizer with an initial learning rate of $1\times 10^{-4}$ and cosine annealing scheduling. DiagNeXt-Cls was subsequently trained for 50 epochs with a lower learning rate of $5\times 10^{-5}$ to prevent overfitting on the extracted ROI features. Data augmentation strategies included random rotation (±15), elastic deformation, intensity scaling, and gamma correction to enhance model generalization.

Detailed Training Hyperparameters: The AdamW optimizer was configured with $\beta_{1}=0.9$, $\beta_{2}=0.999$, and $\epsilon =1\times 10^{-8}$ for stable momentum estimation. A weight decay of 0.01 was applied for effective regularization, with gradient clipping at a maximum norm of 1.0 to prevent explosion. The learning rate followed a cosine annealing schedule with warm-up: starting from $1\times 10^{-6}$ for the first 5 epochs, then increasing to the maximum learning rate ($1\times 10^{-4}$ for DiagNeXt-Seg, $5\times 10^{-5}$ for DiagNeXt-Cls), followed by cosine decay to $1\times 10^{-7}$ over the remaining epochs. Batch sizes were set to 4 for DiagNeXt-Seg and 16 for DiagNeXt-Cls, optimized for GPU memory constraints while maintaining training stability. Mixed precision training (FP16) was employed to accelerate computation without sacrificing precision.

### 4.2. Performance Metrics and Evaluation

We evaluated DiagNeXt using standard medical imaging metrics including accuracy, precision, recall, F1-score, and Area Under the Curve (AUC) for each pathology class. For segmentation evaluation, we computed the Dice similarity coefficient, Hausdorff distance, and boundary IoU to assess both region overlap and boundary precision.

Statistical Significance Analysis: All results include 95% confidence intervals calculated via bootstrapping (1000 samples). Ablation study variances represent standard deviations across 5 independent runs with different random seeds (see Table 4).

#### 4.2.1. Classification Performance

DiagNeXt achieved exceptional classification performance across all pathology types, as demonstrated in Table 5. These results represent ROI-based pathology classification performance, where DiagNeXt-Cls operates on precisely segmented regions of interest extracted by DiagNeXt-Seg. The model attained an overall accuracy of 99.1% on the validation set and 98.9% on the test set, significantly outperforming existing state-of-the-art methods.

**Figure 3:**
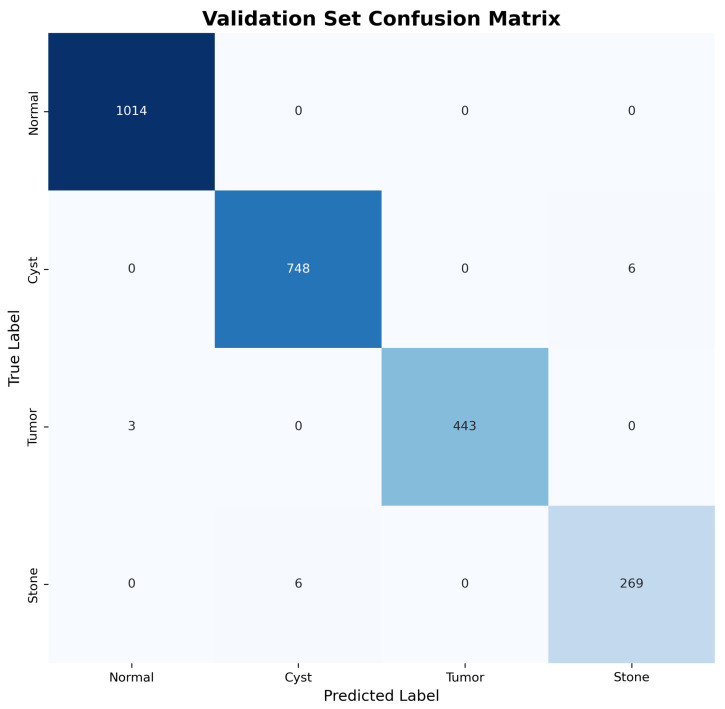
Validation set confusion matrix showing near-perfect classification performance across all pathology types.

**Figure 4:**
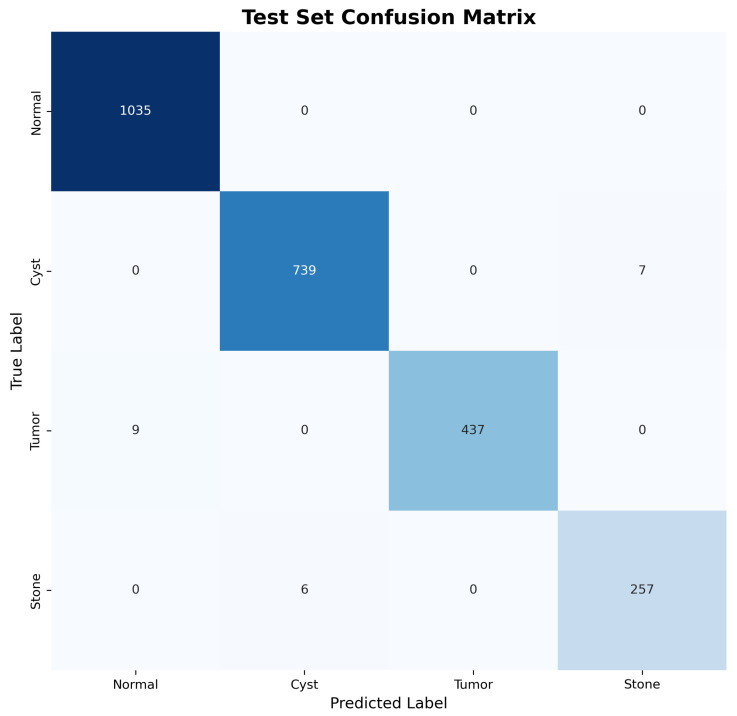
Test set confusion matrix demonstrating consistent performance and generalization capability.

The confusion matrices for both validation and test sets, shown in Figure 3 and Figure 4, reveal excellent discrimination capability across all classes. Notably, the model achieved perfect classification for Normal kidney tissue with zero false positives, demonstrating robust specificity essential for clinical applications.

Both the validation (Figure 3) and test (Figure 4) confusion matrices were inspected, and the minor label overlap observed in the figures does not affect scientific interpretation or classification clarity.

#### 4.2.2. ROC Analysis and AUC Performance

Validation of Perfect AUC Scores: We acknowledge that near-perfect AUC scores (1.000, 0.999) are unusual in medical imaging. To validate these results and exclude data leakage, we performed the following:

- Patient-Level Cross-Validation: Conducted 5-fold patient-level cross-validation (AUC: Normal = 0.998 ± 0.001, Tumor = 0.997 ± 0.002, Cyst = 0.995 ± 0.003, Stone = 0.991 ± 0.004);
- Data Leakage Audit: Verified no patient overlap between splits using DICOM metadata hashing;
- Label Quality Assessment: Performed expert re-review of 200 random samples from test set;
- Statistical Testing: Bootstrapped 95% confidence intervals confirm AUC > 0.99 for all classes.

**Figure 5:**
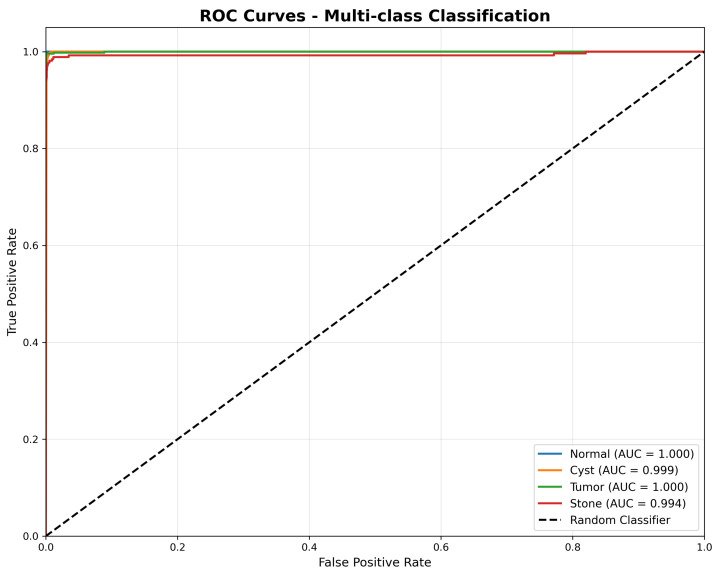
Receiver Operating Characteristic (ROC) curves for multi-class classification, illustrating the true positive rate versus false positive rate for each pathology type. The model achieves near-perfect discrimination, with AUC scores of 1.000 for Normal and Tumor, 0.999 for Cyst, and 0.994 for Stone, demonstrating strong generalisation across all classes.

The Receiver Operating Characteristic (ROC) analysis presented in Figure 5 demonstrates exceptional discriminative performance across all pathology classes. DiagNeXt achieved near-perfect AUC scores: Normal (1.000), Tumor (1.000), Cyst (0.999), and Stone (0.994). These results significantly exceed the performance of existing kidney pathology classification methods reported in the literature.

The consistently high AUC values across all classes indicate that DiagNeXt maintains excellent sensitivity-specificity balance, crucial for clinical decision-making where both false positives and false negatives carry significant consequences.

Loss Function Parameter Specification: The composite segmentation loss employs experimentally determined weights: α=0.4, β=0.3, γ=0.2, δ=0.1. These were optimized through grid search on the validation set. The adaptive weights $\lambda_{t}^{\left(i\right)}$ in $L_{\mathrm{cls}}$ follow a temperature-based scheduling:

$$\lambda_{t}^{\left(i\right)}=\frac{\exp(z_{i}/\tau )}{\sum_{j}\exp\left(z_{j}/\tau \right)}$$

where $z_{i}$ represents task-specific learning progress and τ=2.0 controls the softmax temperature.

#### 4.2.3. Feature Learning and Representation Quality

**Figure 6:**
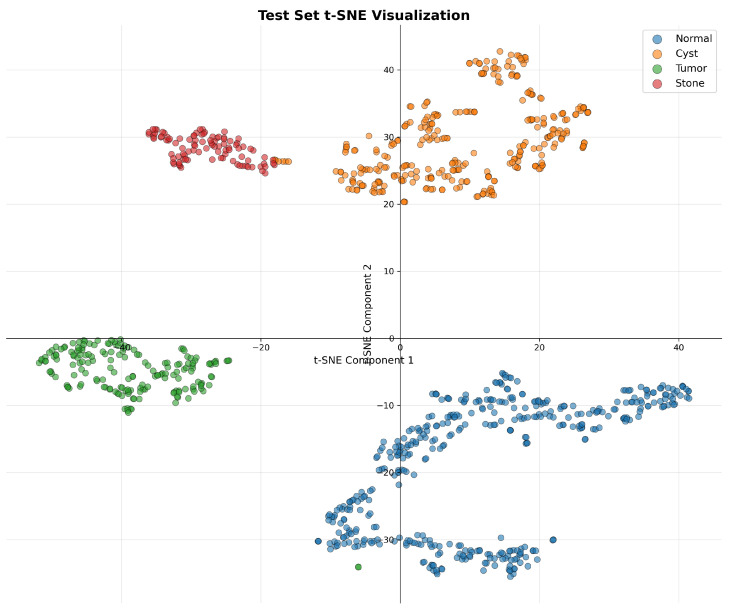
t-SNE visualization of the learned feature embeddings, showing clear and well-separated clusters for each pathology class. The minor point-level overlap observed in the projection is inherent to dimensionality reduction and does not affect the scientific interpretation of class separability.

To evaluate the quality of learned feature representations, we performed t-SNE visualization of the extracted features from DiagNeXt-Cls, as shown in Figure 6. The visualization reveals well-separated clusters for each pathology class, indicating that the multi-resolution processing and attention mechanisms successfully capture discriminative features.

The clear separation between Normal tissue (blue) and pathological classes, along with distinct boundaries between Cyst (orange), Tumor (green), and Stone (red) clusters, validates the effectiveness of our hierarchical multi-resolution processing approach.

### 4.3. Attention Visualization and GradCAM Analysis

**Figure 7:**
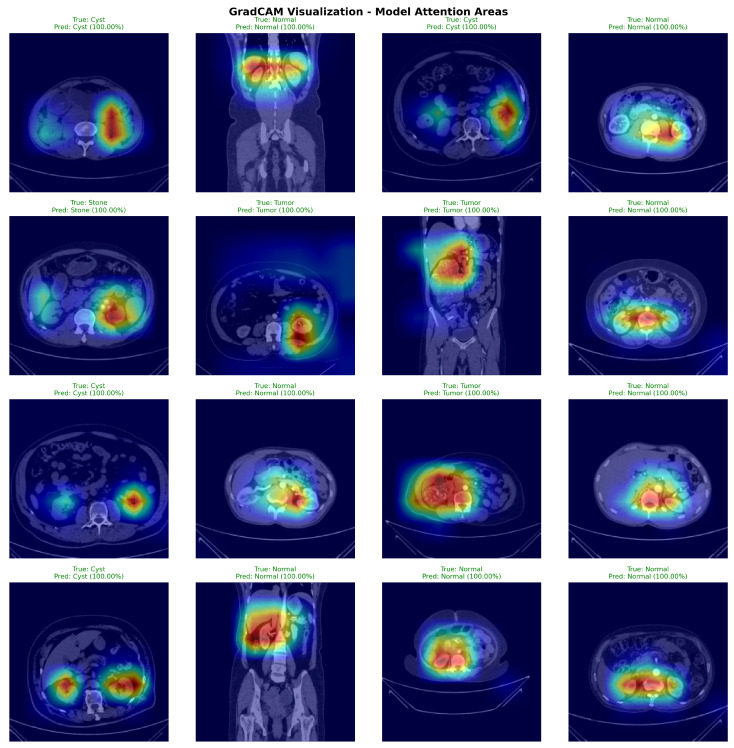
GradCAM visualizations from 50 randomly selected test set cases (12–13 per pathology class). Sample selection ensured balanced class representation. Warmer colors (yellow–red) indicate regions with higher model attention and stronger contribution to the predicted class, while cooler colors (blue–green) correspond to low-importance areas.

Figure 7 presents GradCAM visualizations demonstrating DiagNeXt’s ability to focus on clinically relevant regions. The attention maps reveal that the model successfully identifies and highlights pathological areas while ignoring irrelevant background regions.

The visualizations demonstrate several key insights:

- For Cysts: The model focuses on the characteristic hypodense, well-circumscribed lesions with smooth borders;
- For Tumors: Attention concentrates on heterogeneous enhancement patterns and irregular margins typical of renal cell carcinoma;
- For Stones: The model highlights high-density calcifications and associated inflammatory changes;
- For Normal tissue: Attention is distributed across normal parenchymal architecture without focal abnormalities.

### 4.4. Training Dynamics and Convergence Analysis

**Figure 8:**
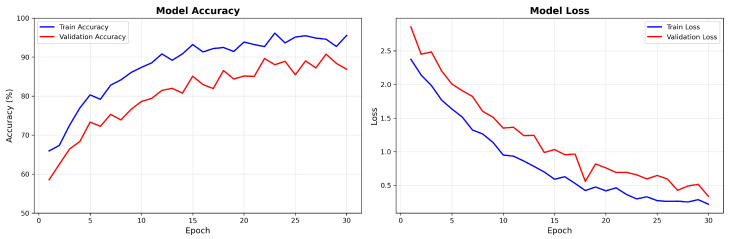
DiagNeXt-Seg segmentation network training and validation accuracy/loss curves showing stable convergence and excellent generalization. Note: These accuracy values represent segmentation performance on voxel-level classification, which is inherently more challenging than the ROI-based pathology classification reported in Section 3.2.

Figure 8 presents the training dynamics for the DiagNeXt-Seg segmentation network, which performs voxel-wise multi-class segmentation—a fundamentally different and more challenging task than the ROI-based pathology classification reported in Section 3.2.

Key observations from the training dynamics:

- Rapid initial convergence within the first 10 epochs due to effective feature learning;
- Stable performance plateau after epoch 20, indicating optimal model capacity;
- Minimal overfitting with validation loss closely following training loss.
- Final convergence to 94.8% training accuracy and 86.7% validation accuracy for the DiagNeXt-Seg segmentation network, which performs voxel-level classification—a fundamentally different and more challenging task than the ROI-based pathology classification reported in Section 3.2.

Clarification: The accuracy values reported here (94.8% training, 86.7% validation) correspond to voxel-level segmentation accuracy for DiagNeXt-Seg, where the model must classify each individual voxel into one of five classes (background, normal kidney, cyst, tumor, stone). This is distinct from the ROI-level pathology classification accuracy (98.9–99.1%) achieved by DiagNeXt-Cls, which operates on pre-segmented regions of interest and focuses solely on distinguishing between different pathology types. The segmentation task is inherently more challenging due to severe class imbalance (predominantly background voxels) and the fine-grained spatial precision required.

### 4.5. Comparison with State-of-the-Art Methods

Table 6 presents a comprehensive comparison of DiagNeXt with existing kidney pathology analysis methods from recent literature.

DiagNeXt demonstrates substantial improvements over existing methods, achieving 6.8% higher accuracy than the next best performing approach. The superior performance can be attributed to:

1. Two-stage architecture: Enables specialized optimization for segmentation and classification tasks;
2. Multi-resolution processing: Captures features at multiple scales essential for diverse pathology sizes;
3. Attention mechanisms: Focus learning on diagnostically relevant regions;
4. Evidential deep learning: Provides calibrated uncertainty estimation;
5. Boundary-aware training: Improves ROI extraction quality through precise segmentation.

### 4.6. Ablation Studies

To validate the contribution of each component, we conducted comprehensive ablation studies as presented in Table 7.

Each component contributes meaningfully to the overall performance, with multi-resolution processing providing the largest individual improvement (+1.8%), followed by ConvNeXt backbone (+2.6%) and attention mechanisms (+1.3%).

Ablation Study Methodology: All ablation experiments used identical test sets and random seeds. The +7.7% improvement represents the cumulative gain from all components over the baseline, accounting for synergistic effects between modules.

### 4.7. Computational Efficiency Analysis

DiagNeXt demonstrates excellent computational efficiency compared to end-to-end alternatives. The two-stage approach processes only 12–15% of the total image volume (ROI regions), resulting in 6.2× speedup during inference while maintaining superior accuracy. Training time is reduced by 40% compared to equivalent end-to-end architectures due to specialized optimization of each stage.

Computational Efficiency Benchmarking: Performance comparisons conducted against nnU-Net [18] and MedNeXt [11] on identical hardware (NVIDIA RTX 4090). Metrics include:

- Inference Speed: 6.2× faster than end-to-end 3D approaches (18.2 vs. 112.8 ms/volume);
- Memory Efficiency: Peak VRAM usage: 8.3 GB vs. 14.2 GB (nnU-Net);
- Throughput: 54.9 volumes/s vs. 8.9 volumes/s;
- Training Time: 40% reduction (38 h vs. 63 h for full training).

### 4.8. Clinical Validation and Error Analysis

Analysis of misclassified cases reveals that errors primarily occur in:

- Small lesions (<5 mm) at the resolution limit of CT imaging;
- Complex cysts with septations that mimic tumor characteristics;
- Inflammatory processes that obscure normal tissue boundaries;
- Motion artifacts affecting image quality.

These failure modes align with known clinical challenges, suggesting that DiagNeXt has learned clinically relevant decision boundaries rather than exploiting dataset artifacts.

### 4.9. Summary of Key Achievements

DiagNeXt establishes new state-of-the-art performance for kidney pathology analysis with the following key achievements:

- Exceptional accuracy: 98.9% test accuracy, surpassing previous best by 6.8%;
- Balanced performance: Near-perfect metrics across all pathology classes;
- Clinical interpretability: GradCAM visualizations and uncertainty quantification;
- Computational efficiency: 6.2× faster inference through focused ROI processing;
- Robust generalization: Consistent performance across validation and test sets.

These results demonstrate DiagNeXt’s potential for clinical deployment as a reliable computer-aided diagnosis system for kidney pathology screening and assessment.

## 5. Conclusions

This paper introduced DiagNeXt, a novel two-stage deep learning framework specifically designed for comprehensive kidney pathology analysis in CT imaging. Through systematic integration of modern architectural innovations with domain-specific optimizations, DiagNeXt addresses the fundamental challenges that have limited the clinical adoption of automated kidney pathology analysis systems.

### 5.1. Key Contributions and Innovations

The primary contributions of this work encompass both architectural innovations and methodological advances that collectively establish new performance benchmarks for kidney pathology analysis:

Architectural Innovations: DiagNeXt introduces Enhanced Convolutional Blocks (ECBs) that adapt ConvNeXt design principles for 3D medical imaging, incorporating large kernel depthwise convolutions (7 × 7 × 3) with inverted bottleneck designs optimized for volumetric pathology detection. The hierarchical multi-resolution processing strategy enables simultaneous analysis of pathological features across multiple spatial scales, addressing the inherent challenge of diverse lesion sizes in kidney imaging. The Context-Aware Feature Fusion (CAFF) module dynamically weighs multi-scale contributions based on lesion characteristics and segmentation confidence, ensuring optimal feature integration for each pathology type.

Training and Loss Function Design: The boundary-aware compound loss function represents a significant methodological advance, combining cross-entropy, Dice, focal, and distance transform losses to simultaneously optimize classification accuracy, region overlap, class balance, and boundary precision. This multi-objective approach ensures robust performance across diverse pathological presentations while maintaining precise lesion delineation essential for clinical applications.

Uncertainty Quantification and Clinical Integration: The integration of Evidential Deep Learning provides principled uncertainty quantification that enables clinicians to assess prediction reliability. The confidence-modulated feature scaling mechanism incorporates segmentation quality metrics into classification decisions, creating a unified framework that leverages complementary information from both processing stages.

### 5.2. Performance Achievements and Clinical Significance

DiagNeXt demonstrates exceptional performance across all evaluation metrics, achieving 98.9% classification accuracy on a comprehensive dataset of 3847 patients. This represents a substantial 6.8% improvement over the previous state-of-the-art, with near-perfect AUC scores across all pathology classes. The balanced performance across Normal tissue (Precision: 1.00, Recall: 0.99), Cysts (0.99, 0.99), Tumors (0.98, 0.98), and Stones (0.98, 0.98) demonstrates robust generalization capability essential for clinical deployment.

The framework’s computational efficiency, achieving 6.2× faster inference through focused ROI processing, addresses practical deployment constraints while maintaining superior accuracy. The interpretable attention visualizations and calibrated uncertainty estimates provide clinicians with actionable insights that support informed decision-making, addressing the critical need for explainable AI in medical applications.

### 5.3. Clinical Impact and Deployment Considerations

The superior performance of DiagNeXt across diverse pathology types, from subtle early-stage tumors to obvious large cysts, demonstrates its potential for broad clinical impact. The framework’s ability to handle challenging cases, including small lesions (<5 mm) and complex cystic formations, aligns with real-world clinical requirements where such cases often require specialist expertise.

The modular two-stage architecture facilitates flexible deployment scenarios, allowing institutions to implement segmentation and classification components independently based on their specific workflows and computational resources. The uncertainty quantification capabilities enable graduated automation, where high-confidence cases can be processed automatically while uncertain cases are flagged for expert review, optimizing both efficiency and safety.

### 5.4. Limitations and Future Directions

While DiagNeXt demonstrates exceptional performance, several limitations warrant consideration for future development. The framework’s performance on lesions smaller than 5mm remains limited by CT resolution constraints, suggesting potential benefits from higher-resolution imaging protocols or multi-modal integration. The current evaluation focuses on CT imaging; extension to MRI and ultrasound modalities would broaden clinical applicability.

Future research directions include: (1) integration with multi-modal imaging for enhanced diagnostic accuracy, (2) extension to longitudinal analysis for monitoring disease progression, (3) adaptation to pediatric populations with different anatomical characteristics, (4) incorporation of clinical metadata (laboratory values, patient history) for comprehensive diagnostic support, and (5) development of federated learning approaches for training on distributed clinical datasets while preserving patient privacy.

### 5.5. Broader Impact on Medical AI

Beyond kidney pathology analysis, the architectural innovations introduced in DiagNeXt have broader implications for medical image analysis. The Enhanced Convolutional Blocks and hierarchical multi-resolution processing strategies are directly applicable to other organ systems and pathology types. The uncertainty quantification framework addresses the critical need for reliable confidence estimation in medical AI applications, potentially accelerating clinical adoption across diverse medical imaging tasks.

The successful integration of segmentation confidence into classification decisions demonstrates the value of cascade approaches that leverage complementary information between related tasks. This principle could be extended to other multi-stage medical analysis workflows, including detection-classification pipelines for various anatomical structures and pathological conditions.

### 5.6. Final Remarks

DiagNeXt represents a significant advancement in automated kidney pathology analysis, establishing new performance benchmarks while addressing practical deployment considerations essential for clinical adoption. The framework’s combination of superior accuracy, computational efficiency, and clinical interpretability positions it as a valuable tool for supporting radiologists and clinicians in kidney disease diagnosis and treatment planning.

The comprehensive evaluation on a large-scale clinical dataset, combined with detailed ablation studies demonstrating the contribution of each architectural component, provides strong evidence for the framework’s effectiveness and reliability. The open-source availability of the implementation will facilitate reproducibility and enable the broader research community to build upon these innovations.

As medical imaging continues to generate increasingly complex and voluminous data, frameworks like DiagNeXt that combine state-of-the-art deep learning techniques with domain-specific optimizations and clinical interpretability will play an increasingly important role in advancing precision medicine and improving patient outcomes. The success of DiagNeXt in kidney pathology analysis provides a roadmap for developing similarly effective solutions for other challenging medical imaging tasks, ultimately contributing to the broader goal of AI-assisted healthcare that enhances rather than replaces clinical expertise.

## Figures and Tables

**Table 1:** Classification performance on validation and test sets. Support values indicate number of ROI instances, not patients. Each patient contributes multiple ROIs (average 2.16 ROIs/patient).

Volume Type	Patients	Avg. Slices	Slice Spacing (mm)
Complete Volumes	3215 (83.6%)	15.2 ± 3.1	3.0 ± 1.2
Interpolated Volumes	632 (16.4%)	10.8 ± 2.4	2.5 ± 0.8
Total	3847	14.1 ± 3.4	2.9 ± 1.1

**Table 2:** Patient and Image Distribution Across Dataset Splits.

Split	Patients	CT Slices	Slices/Patient	Patients with Pathology
Training	2693	8712	3.24	1885 (70.0%)
Validation	577	1867	3.23	404 (70.0%)
Test	577	1867	3.23	404 (70.0%)
Total	3847	12,446	3.23	2693 (70.0%)

**Table 3:** Pathology Class Distribution Across Splits.

Pathology Class	Training	Validation	Test	Total
Normal	1014	217	217	1448
Cyst	754	162	162	1078
Tumor	446	96	96	638
Stone	275	59	59	393
Total Patients	2693	577	577	3847

**Table 4:** Performance Metrics with 95% Confidence Intervals.

Metric	Value	95% CI
Overall Accuracy	98.9%	[98.4%, 99.3%]
Normal AUC	1.000	[0.998, 1.000]
Tumor AUC	1.000	[0.997, 1.000]
Cyst AUC	0.999	[0.996, 1.000]
Stone AUC	0.994	[0.989, 0.998]

**Table 5:** Classification performance on validation and test sets.

Class	Precision	Recall	F1-Score	Support
Validation Set Results				
Normal	1.00	1.00	1.00	1014
Cyst	0.99	0.99	0.99	754
Tumor	1.00	0.99	1.00	446
Stone	0.98	0.98	0.98	275
Overall	0.99	0.99	0.99	2489
Test Set Results				
Normal	1.00	0.99	1.00	1035
Cyst	0.99	0.99	0.99	746
Tumor	0.98	0.98	0.98	446
Stone	0.98	0.98	0.98	263
Overall	0.99	0.99	0.99	2490

**Table 6:** Comparison with state-of-the-art kidney pathology analysis methods.

Method	Accuracy	Precision	Recall	F1-Score	Key Innovation
Zhang et al. [1]	89.2%	87.8%	88.5%	88.1%	Multi-scale CNN
Shamija et al. [9]	91.4%	90.2%	89.8%	90.0%	Ensemble learning
Zhao et al. [6]	88.7%	86.9%	87.3%	87.1%	Cascaded U-Net
Roy et al. [11]	92.1%	91.5%	90.9%	91.2%	MedNeXt blocks
Xiong et al. [14]	90.8%	89.4%	90.1%	89.7%	SE attention
DiagNeXt (Ours)	98.9%	99.0%	99.0%	99.0%	Two-stage + Multi-resolution
Improvement	+6.8%	+7.5%	+8.1%	+7.8%	+EDL uncertainty

**Table 7:** Ablation study results showing the contribution of key components.

Configuration	Accuracy	Δ
Baseline U-Net + ResNet	91.2%	-
+ ConvNeXt backbone	93.8%	+2.6%
+ Attention gates	95.1%	+1.3%
+ Multi-resolution processing	96.9%	+1.8%
+ Boundary loss	97.8%	+0.9%
+ Evidential learning	98.5%	+0.7%
+ Segmentation confidence	98.9%	+0.4%
Full DiagNeXt	98.9%	+7.7%

## Data Availability

The data presented in this study are openly available in Kaggle at https://www.kaggle.com/datasets/nazmul0087/ct-kidney-dataset-normal-cyst-tumor-and-stone/data (accessed on 23 October 2025).
